# Exploring knowledge, attitude, and intention towards advance care planning, advance directive, and the patient self-determination act among hemodialysis patients

**DOI:** 10.1186/s12904-023-01321-2

**Published:** 2023-12-14

**Authors:** Shang-Feng Tsai, Ching-Yi Chang, Jia-Yi Yang, Yu-Ying Ho, Ching-Ching Hsiao, Shu-Chuan Hsu, Shih-Yun Chen, Huan-Yi Lin, Te-Feng Yeh, Cheng-Hsu Chen

**Affiliations:** 1 Division of Nephrology, Department of Internal Medicine, Taichung Veterans General Hospital, 160, Sec. 3, Taiwan Boulevard, Taichung, 407 Taiwan; 2 Department of Life Science, Tunghai University, Taichung, Taiwan; 3 Department of Post-Baccalaureate Medicine, College of Medicine, National Chung Hsing University, Taichung, Taiwan; 4 Department of Nursing, Taichung Veterans General Hospital, Taichung, Taiwan; 5 Department of Ophthalmology, Taichung Veterans General Hospital, Taichung, Taiwan; 6 Department of Healthcare Administration, Central Taiwan University of Science and Technology, Taichung, Taiwan; 7 Ph.D. Program in Tissue Engineering and Regenerative Medicine, College of Medicine, National Chung Hsing University, Taichung, Taiwan

**Keywords:** Advance directive (AD), Advance care planning (ACP), Patient self-determination act (PSDA), Life-sustaining treatment (LST), Hemodialysis, Knowledge, Attitude, Intention, Life-sustaining treatment, Patient right to autonomy act

## Abstract

**Background:**

Hemodialysis holds the highest incidence and prevalence rate in Taiwan globally. However, the implementation of advance care planning (ACP), advance directives (AD), and patient self-determination acts (PSDA) remains limited. Our objective was to examine the current status of ACP, AD and PSDA and potential opportunities for enhancement.

**Methods:**

We developed a novel questionnaire to assess individuals’ knowledge, attitudes, and intentions regarding ACP, AD, and PSDA. We also collected baseline characteristics and additional inquiries for correlation analysis to identify potential factors. Student’s t-test and Analysis of Variance were employed to assess significance.

**Results:**

Initially, a cohort of 241 patients was initially considered for inclusion in this study. Subsequently, 135 patients agreed to participate in the questionnaire study, resulting in 129 valid questionnaires. Among these respondents, 76 were male (59.9%), and 53 were female (41.1%). Only 13.2% had signed AD. A significant portion (85.3%) indicated that they had not discussed their dialysis prognosis with healthcare providers. Additionally, a mere 14% engaged in conversations about life-threatening decisions. Ninety percent believed that healthcare providers had not furnished information about ACP, and only 30% had discussed such choices with their families. The findings revealed that the average standardized score for ACP and AD goals was 84.97, while the attitude towards PSDA received a standardized score of 69.94. The intention score stood at 69.52 in standardized terms. Potential candidates for ACP initiation included individuals aged 50 to 64, possessing at least a college education, being unmarried, and having no history of diabetes.

**Conclusion:**

Patients undergoing hemodialysis exhibited a significant knowledge gap concerning ACP, AD, and the PSDA. Notably, a substantial number of dialytic patients had not received adequate information on these subjects. Nevertheless, they displayed a positive attitude, and a considerable proportion expressed a willingness to sign AD. It is imperative for nephrologists to take an active role in initiating ACP discussions with patients from the very beginning.

**Supplementary Information:**

The online version contains supplementary material available at 10.1186/s12904-023-01321-2.

## Introduction

The incidence of dialysis had previously shown a plateau, contrasting with an escalation in hemodialysis withdrawal rates [1]. However, there has been a growing prevalence of dialysis cases in Taiwan [2], while the withdrawal rates in the country remain limited [3]. In the Taiwanese context, barriers persist in providing hospice care and ceasing dialysis for patients with advanced renal failure [3–5]. Notably, both the incidence of chronic kidney disease (CKD) [6, 7] and the prevalence of end-stage kidney disease (ESKD) [8, 9] are notably higher in Taiwan compared to other nations. One contributing factor to the high prevalence of dialysis patients is the infrequent withdrawal or withholding of dialysis.

Hemodialysis offers life extension benefits, yet it carries inherent risks like infection, sudden death, and patient distress. Determining the appropriate timing for discontinuing hemodialysis relies on patients’ medical status, physician guidance, and patient preferences. Preferences and decision-making processes differ between Taiwan and Western countries. The unique cultural aspects in Taiwan [3] introduce distinct considerations, such as increased family involvement in decision-making [10, 11], withholding negative news from patients [12], and active engagement in a patient’s end-of-life care [13–15]. Notably, family involvement is pivotal, shaping a decision-making model centered around families and often discouraging the disclosure of disease-related information. However, the ultimate treatment decisions should be driven by patients rather than adhering strictly to a paternalistic model. Extensive literature reviews suggest that advance care planning (ACP) and palliative care do not evoke discomfort or anxiety in dialysis patients [16, 17]. Moreover, dialysis patients exhibit high comfort levels with the communication process.

ACP encompasses the process of making future medical decisions and expressing patient preferences when they have a comprehensive understanding of their medical condition. Despite the advocacy by the Renal Physicians Association for ACP among ESKD patients, variations in cultural perceptions exist across different countries [18–23]. In Taiwan, there is limited information available on ACP, advance directives (AD), and patient self-determination acts (PSDA), necessitating an examination of the underlying reasons. In our previous study [24], CKD patients scored the highest in health literacy across five dimensions (access, understanding, appraising, applying health information, and communication). They exhibited proficiency in accessing and comprehending health information. However, the proficiency in ACP, AD, and PSDA might differ. While health literacy encompasses aspects related to ‘health’ and ‘quality of life,’ ACP, AD, and PSDA are focused on the “quality of end-of-life care”. Moreover, the inclination towards engaging or abstaining from ACP, AD, and PSDA is primarily rooted in emotional responses. However, research exploring these emotional responses remains relatively scarce. Therefore, this study was undertaken to assess the current status of ACP, AD, and PSDA in hemodialysis patients and to identify potential contributing factors.

## Materials and methods

### Definition of population

This research conducted a survey among hemodialysis patients at Taichung Veterans General Hospital in Taiwan in 2019. The inclusion criteria encompassed patients actively receiving hemodialysis treatment, possessing the ability to provide coherent responses, proficiency in Mandarin or Taiwanese languages, and having provided signed informed consent. Patients unable to respond autonomously were excluded from participation. The study received approval from the Institutional Review Board of Taichung Veterans General Hospital (Approval No: CE19142B).

### Study design

The study’s design framework is depicted in Supplementary Fig. 1. Employing a questionnaire-based approach, we investigated patients’ knowledge and attitudes toward ACP and AD. Additionally, we evaluated intentions concerning the PSDA, encompassing the signing of PSDA and life-sustaining treatment (LST) documents. Various dimensions of variables, including demographics, hemodialysis-related factors, and other pertinent experiences, were collected to explore their associations with knowledge, attitudes, and intentions regarding ACP, AD, and PSDA. Our objective was to ascertain potential correlations between attitudes and knowledge, determine the impact of knowledge on intentions, and identify any associations between knowledge and intentions.

The research questionnaire addresses topics related to death. After providing an explanation of the questionnaire’s content and obtaining patient consent, distribution and completion of the questionnaire occurred during routine dialysis sessions. In instances where a patient was unable to complete the questionnaire independently, the researcher provided assistance through inquiry.

### Design of questionnaire

Recent studies conducted in Taiwan [25, 26] have highlighted variations in nurses’ knowledge, attitudes, and practice behaviors toward ACP. These studies [25, 26] utilized a 34-item measurement tool based on Zhou’s research [27]. However, our study redirected its focus towards patients and developed a structured questionnaire tailored for this purpose. The questionnaire’s content was derived from relevant literature sources [28]. Subsequently, twelve scholars with practical experience were invited to evaluate the questionnaire’s expert validity (see Supplementary Table 1). They reviewed the content, assessed semantic accuracy, appropriateness, and provided ratings, alongside guidance and suggestions for revisions.

The questionnaire encompasses five primary sections: patient background information (19 questions), knowledge of ACP and AD (10 questions), attitude toward ACP and AD (41 questions), the importance of ACP (10 questions), and intention toward ACP and PSDA (12 questions). Additionally, there are six other related questions concerning ACP and PSDA. The operational definitions of patient basic characteristics and other associated questions are outlined in Supplementary Tables 2 and 3, respectively. Detailed information about the questionnaire is available in Supplementary Table 4. The complete questionnaire contents are accessible in Supplementary Table 5 (translated into English). The questionnaire employs a Likert scale for scoring, ranging from 1 (‘strongly agree’) to 5 (‘strongly disagree’) with the statements in the questionnaire.

Before administration to all patients, the questionnaire underwent a pre-test to evaluate its reliability and validity. Regarding validity, twelve experts from academia and the field reviewed the questionnaire’s content, as detailed in Supplementary Table 1. The Content Validity Index (CVI) was calculated at 0.976. A pilot test involving 33 hemodialysis patients was conducted to assess the questionnaire’s clarity, comprehensibility, and completion time. In terms of reliability, Cronbach’s α coefficient was utilized to measure the questionnaire’s internal consistency for each dimension. The pre-test knowledge indicated a Cronbach’s α of 0.916, which increased to 0.956 in the formal test. Pre-test attitudes ranged from 0.983 to 1.000, while in the formal test, it ranged from 0.911 to 0.983. The Cronbach’s α for pre-test importance was 0.916, rising to 0.985 in the formal test. Pre-test intention varied from 0.950 to 0.976, and in the formal test, it ranged from 0.843 to 0.962 (as presented in Supplementary Table 6).

### Statistical analyses

Descriptive statistics were employed to present participants’ demographic characteristics, while means with standard deviations were used for variables including knowledge, attitude, and intention. Standardization for knowledge and intention was conducted using the formula [(sum of scores / maximum possible score) × 100]. Age was categorized into four groups: ‘Below 49 years,’ ‘50–64 years,’ ‘65–74 years,’ and ‘75 years and above’. Treatment duration was classified as ‘Less than 5 years,’ ‘6–10 years,’ and ‘More than 10 years’. Due to constraints in sample size, education levels were consolidated into three categories: ‘Junior high school and below,’ ‘High school,’ and ‘College and above’. Marital status was merged into ‘Married,’ ‘Unmarried,’ and ‘Separated, Divorced, Widowed’. Religious beliefs were grouped as ‘No religious beliefs,’ ‘Buddhist/Taoist,’ and ‘Christian/Catholic’. Preferences for end-of-life discussions were combined into ‘Don’t want to discuss,’ ‘Nephrologist,’ and ‘Family members’. Opinions on ACP initiators were merged into ‘Patient themselves,’ ‘Nephrologist,’ and ‘Others’.

To evaluate differences in knowledge, attitude, and intention regarding ACP and PSDA among hemodialysis patients based on demographic factors, Student’s t-test and Analysis of Variance (ANOVA) were utilized. Statistical significance was determined at a p-value of < 0.05, indicating noteworthy differences or associations between variables. All statistical analyses were conducted using IBM SPSS statistical software version 25.0 (Chicago, IL) to ensure accuracy and reliability of the outcomes.

## Results

### Baseline characteristics of this cohort

The baseline characteristics of the cohort are summarized in Table 1. Initially, the study considered 241 patients; however, ultimately, 129 valid responses were obtained. Among these participants, 76 (59.9%) were male, with an average age of 61.69 years. The most prevalent age group was 59–64 years, accounting for 45.7% of the cohort. Regarding educational attainment, 37.2% completed junior high school, 32.6% held a college degree or higher, and 30.2% had education levels below junior high school. Roughly 70% of the participants were married, and 66.7% identified with Buddhist or Taoist religious beliefs. The majority of patients resided with their families (92.2%).

**Table 1:** Baseline characteristics of this cohort

	N	Percentage (%)	Mean	Standard deviation
Gender				
Male	76	58.9		
Female	53	41.1		
Age (years old)			61.69	13.14
≥ 49	20	15.5		
50 ~ 64	59	45.7		
65 ~ 74	25	19.4		
≥ 75	25	19.4		
Level of education				
Below Junior High School	39	30.2		
Junior High School	48	37.2		
College and above	42	32.6		
Marriage				
Married	98	76.0		
Single	20	15.5		
Divorced, Separated, Widowed	4	3.1		
Religion				
No	33	25.6		
Buddhism or Taoism	86	66.7		
Roman Catholicism or Christianity	10	7.8		
Residential Status				
Living Alone	8	6.2		
Living with Family	119	92.2		
Living with Others	2	1.6		
Financial Status				
Sufficient work income to support	45	34.9		
Sufficient savings or retirement funds to support	34	26.4		
Depend on family and friends for support	43	33.3		
Depend on social assistance for support	7	5.4		
Comorbidity				
Hypertension	75	58.1		
Diabetes mellitus	52	40.3		
Cardiovascular disease	33	25.6		
Arrhythmia	20	15.5		
Dyslipidemia	13	10.1		
Heart failure	5	3.9		
Peptic ulcer disease	5	3.9		
Malignancy	5	3.9		
Ischemic heart disease	4	3.1		
Stroke	4	3.1		
Others	19	14.7		
Times of hemodialysis per week				
2	8	6.2		
3	121	93.8		
Vintage of hemodialysis (years)			6.98	6.62
< 5	59	45.7		
5 ~ 9	37	28.7		
≥ 1	33	25.6		
The main reasons for choosing the dialysis method				
Doctor’s recommendation	83	64.3		
Personal preference	44	34.1		
Family’s expectations	2	1.6		
(Formerly) Engaged in medical-related work				
Yes	8	6.2		
No	121	93.8		
Feeling confused about the current dialysis decision				
Yes	15	11.6		
No	114	88.4		
Have you signed a Do-Not-Resuscitate (DNR) order or an Advance Care Planning (ACP) and Advance Directive (AD) document for palliative care and life-sustaining treatment decisions?				
Yes	17	13.2		
No	112	86.8		
Have healthcare providers discussed with you and your family about the prognosis and estimated survival time for dialysis?				
Yes	19	14.7		
No	110	85.3		
Have healthcare providers discussed with you or your family about medical decisions when the condition worsens and becomes life-threatening?				
Yes	18	14.0		
No	111	86.0		
Healthcare providers have provided you or your family with information about Advance Care Planning (ACP) and Advance Directives (AD)				
Yes	5	3.9		
No	124	96.1		
Have you ever discussed medical decisions with your family when the condition worsens and becomes life-threatening?				
Yes	40	31.0		
No	89	69.0		

Regarding economic status, 34.9% relied on income from work, 33.3% depended on support from family or friends, and 26.4% had savings. Common coexisting conditions included hypertension (58.1%), diabetes (40.3%), cardiovascular diseases (25.6%), and arrhythmias (15.5%). Most patients (90%) underwent thrice-weekly dialysis, with an average treatment duration of 6.98 years. Reasons for initiating dialysis were primarily influenced by physician recommendations (60%), personal preferences (30%), and family expectations (10%). Furthermore, 93.8% of participants did not have a background in medically related professions, and 88.4% reported no confusion related to their dialysis treatments.

Notably, only 13.2% of participants had signed a Do-Not-Resuscitate (DNR) or Advance Directive (AD). A significant majority (85.3%) had not engaged in discussions regarding their dialysis prognosis with healthcare providers. Merely 14% had conversations regarding life-threatening decisions. Moreover, 90% of participants felt that healthcare providers had not sufficiently addressed Advance Care Planning (ACP), while 30% had discussed it with their families.

### Hemodialysis patients’ knowledge, attitudes, and intentions towards ACP, AD and PSDA

**Fig. 1:**
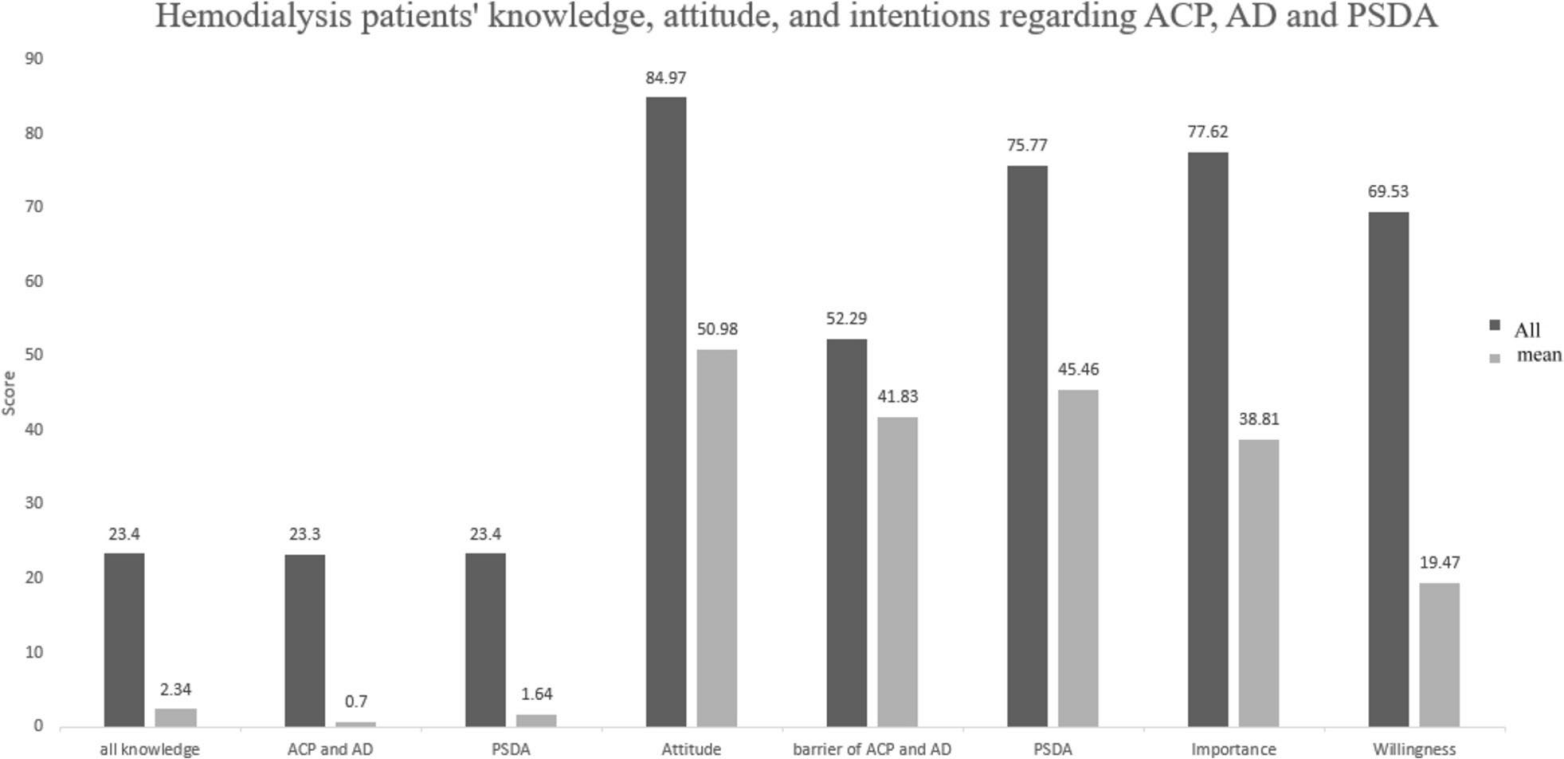
Hemodialysis patients’ knowledge, attitude, and intentions regarding ACP, AD and PSDA

Comprehensive information concerning patient knowledge, attitudes, and intentions toward ACP, AD, and the PSDA is detailed in Supplementary Table 7, with summarized outcomes depicted in Fig. 1. The knowledge assessment comprises two components: ACP and AD knowledge (assessed with 3 questions, scored 0–3) and PSDA knowledge (evaluated with 7 questions, scored 0–7). The mean combined knowledge score is 2.34 out of 10, with ACP and AD knowledge averaging 0.70 and PSDA knowledge scoring 1.64. When standardized to a 100-point scale, the total knowledge score is 23.4, the ACP and AD knowledge score is 23.3, and the PSDA knowledge score is 23.4. Correct answer percentages for both ACP and AD, as well as PSDA knowledge, remain below 30%, with the lowest being ‘Do you know: Medical proxy can be more than one person?’ at 18.6%.

Regarding attitudes, it was divided into four dimensions: ACP and AD goals (13 questions, scored 13–65), ACP and AD barriers (15 questions, scored 15–75), attitude toward PSDA (13 questions, scored 13–65), and ACP importance (10 questions, scored 10–50). Results revealed that the average score for ACP and AD goals was 50.98, standardized to 84.97, with scores ranging between 3.77 and 3.96. Notably, “Believe that discussions about ACP should start early in the initial stage of dialysis for patients with ESKD” had a slightly lower score of 3.77. ACP and AD barriers averaged 41.83, standardized to 52.29, with the item “For patients with ESKD, prolonging life treatment is more important than discontinuing life-sustaining treatment” scoring 3.03. The average attitude toward PSDA was 45.46, standardized to 69.94, with scores spanning 3.67 to 3.21. The item “The medical team has the right to not follow the patient’s AD based on their expertise or preference” scored 3.21. Regarding importance, the average score was 38.81, standardized to 77.62, with “Assist patients in designating a healthcare proxy for future care decisions” and “Help healthcare proxies understand their role and responsibilities in the patient’s future medical decisions” having slightly lower scores of 3.83 each.

Intention was categorized into two parts: intention to sign (7 questions, scored 7–28) and intention for life-sustaining treatment (5 questions, scored 5–20). The average intention to sign score was 19.47, standardized to 69.52, with “Are you willing to encourage family members to also sign AD” scoring 2.74. The standardized intention for life-sustaining treatment was 54.05, with average scores ranging from 1.88 to 2.69. The item “If diagnosed with a specific clinical condition, would you be willing to undergo endotracheal intubation treatment” scored 1.88.

Regarding other questions, the majority of hemodialysis patients (68.2%) favored discussing end-of-life intentions with their family. Concerning ACP timing, a significant majority (47.3%) preferred discussing it during good health, while 18.6% preferred addressing serious complications, and 14.7% opted for kidney function decline. Regarding ACP discussions, 67 patients (51.9%) believed nephrologists should initiate, whereas 50 patients (38.8%) thought patients should. Spouses (72 individuals, 55.8%) and children (64 individuals, 49.6%) were popular surrogate decision-makers. About payment, three-quarters preferred the lowest option of 33 US dollars for ACP. Most (60%) felt national health insurance or government subsidies should cover “ACP consultation fees,” while less than 10% were willing to pay themselves.

### Associations between basic characteristics and knowledge, attitudes, and intentions

Table 2 presents the associations between baseline variables and knowledge, attitude, and intention. Age demonstrated significant differences in total (*p* < 0.05), ACP and AD (*p* < 0.05), and PSDA knowledge (*p* < 0.05). Generally, higher knowledge scores were linked to individuals aged 50–64, higher educational attainment, unmarried status, absence of diabetes, confidence in dialysis decisions, receiving ACP and AD information from healthcare providers, and discussing LST with family. Females exhibited greater importance attitudes toward ACP (*p* < 0.05) than males.

**Table 2:** Associations between basic characteristics and knowledge, attitudes, and intentions

Personal characteristics	All knowledge	Knowledge of ACP and AD	Knowledge of PSDA	Goals of ACP and AD	Barriers to ACP and AD	PSDA	Importance of ACP	Intention to sign	Intention to LST
Gender	-0.294	-0.767	-0.062	-0.582	0.384	-0.866	-2.201^*^	-2.957^**^	0.813
Male	2.26	0.63	1.63	46.75	46.00	45.07	37.75	18.61	10.98
Female	2.45	0.79	1.66	47.53	45.37	46.01	40.32	20.69	10.56
Age^a^	3.234^*^	2.668^*^	3.162^*^	2.748^*^	1.616	3.469^*^	2.663^*^	0.565	1.168
< 49y/o	3.05	1.05	2.00	49.75	42.17	48.02	41.69	20.55	10.65
50 ~ 64y/o	3.10	0.86	2.24	47.93	45.86	46.24	39.35	19.29	11.25
65 ~ 74y/o	0.84	0.24	0.60	44.16	48.17	43.26	37.55	19.24	10.71
≥ 75 y/o	1.48	0.48	1.00	45.79	45.88	43.76	36.48	19.24	9.99
Post Hoc									
Level of education^a^	7.922^***^	4.550^*^	8.626^***^	5.748^**^	4.586^*^	3.283^*^	1.805	2.786	0.360
Below Junior High School	0.95	0.31	0.64	44.07	49.13	43.69	37.27	18.60	10.70
Junior High School	2.08	0.69	1.40	47.42	45.21	45.47	38.89	19.17	10.63
College and above	3.93	1.07	2.86	49.45	43.20	47.07	40.14	20.61	11.12
Post Hoc	3 > 2 > 1	3 > 1	3 > 2 > 1	3 > 1	1 > 3	3 > 1			
Marriage^a^	4.600^*^	6.172^**^	3.556^*^	0.775	1.109	0.835	4.998^**^	2.359	0.369
Married	2.28	0.63	1.64	50.73	42.27	45.23	38.81	19.37	10.79
Single	3.95	1.40	2.55	52.90	39.02	47.02	41.59	20.90	11.20
Divorced, Separated, Widowed	0.00	0.00	0.00	49.63	43.09	44.64	33.73	17.73	10.27
Post Hoc	2 > 3	2 > 1,3	2 > 3				2 > 3		
Religon^a^	0.653	0.626	0.706	3.067	2.097	1.612	1.504	1.092	0.149
No	1.73	0.55	1.18	49.91	39.03	43.85	38.12	18.57	10.97
Buddhism or Taoism	2.53	0.72	1.81	51.97	42.91	46.06	38.66	19.76	10.80
Roman Catholicism or Christianity	2.70	1.00	1.70	45.90	41.80	45.60	42.30	19.87	10.40
Post Hoc									
Residential Status^a^	0.009	0.077	0.062	4.377^*^	0.222	1.267	0.676	0.018	0.342
Living Alone	2.38	0.75	1.63	42.25	47.50	42.38	36.13	19.50	11.38
Living with Family	2.34	0.69	1.66	47.58	45.58	45.62	38.97	19.45	10.75
Living with Others	2.00	1.00	1.00	36.00	48.00	48.00	40.00	20.00	12.00
Post Hoc									
Financial Status^a^	1.315	0.441	1.735	0.635	1.118	0.700	0.740	1.651	3.250^*^
Sufficient work income to support	2.80	0.82	1.98	48.20	43.89	45.23	39.11	19.82	11.07
Sufficient savings or retirement funds to support	2.88	0.74	2.15	45.91	46.89	46.52	39.50	18.38	11.64
Depend on family and friends for support	1.60	0.58	1.02	46.79	46.25	44.66	38.50	20.17	9.76
Depend on social assistance for support	1.29	0.43	0.86	47.14	48.95	46.62	35.43	18.14	11.57
Post Hoc									2 > 3
Comorbidity^t^									
Hypertension	0.689	0.865	0.549	0.424	-2.456^*^	-.392	0.469	0.913	-2.123^*^
Yes	2.52	0.77	1.75	47.31	44.09	45.28	39.05	19.74	10.36
No	2.09	0.59	1.50	46.74	48.04	45.70	38.47	19.08	11.44
Cardiovascular disease	-0.80	-0.348	-0.950	-3.483^***^	0.473	-1.476	-1.886	-2.004^*^	0.110
Yes	1.91	0.64	1.27	43.33	46.39	44.12	36.89	18.26	10.85
No	2.49	0.72	1.77	48.35	45.52	45.91	39.47	19.88	10.80
Diabetes mellitus	-2.076^*^	-2.170^*^	-1.901	-1.148	0.602	0.946	-0.352	-0.687	1.050
Yes	1.58	0.44	1.13	46.15	46.33	46.07	38.55	19.17	11.14
No	2.86	0.87	1.99	47.69	45.34	45.04	38.98	19.67	10.59
Heart failure	0.699	0.979	0.696	2.216^*^	-1.431	-0.398	2.448^*^	1.437	-0.321
Yes	4.00	1.20	2.80	54.20	40.00	44.40	46.00	22.00	10.40
No	2.27	0.68	1.60	46.78	45.97	45.50	38.52	19.36	10.83
Ischemic heart disease	0.089	0.091	0.083	-1.041	-1.273	-3.578^*^	0.651	0.268	-0.154
Yes	2.50	0.75	1.75	43.25	40.00	40.50	41.00	20.00	10.76
No	2.34	0.70	1.64	47.19	45.92	45.62	38.74	19.45	10.81
Dyslipidemia	-0.116	-0.266	-0.041	-1.334	-0.903	-1.089	-1.361	-0.437	0.218
Yes	2.23	0.62	1.62	44.46	43.56	43.73	36.37	19.00	10.98
No	2.35	0.71	1.65	47.36	45.98	45.65	39.08	19.52	10.79
Peptic ulcer disease	0.417	-0.190	0.663	1.020	0.076	0.088	1.483	0.188	0.621
Yes	3.00	0.60	2.40	50.40	46.05	45.69	39.76	19.80	11.60
No	2.31	0.70	1.61	46.93	45.73	45.45	38.77	19.45	10.78
Stroke	0.089	0.091	0.083	1.073	-0.384	0.182	-0.396	-0.736	1.011
Yes	2.50	0.75	1.75	51.00	44.00	46.00	37.47	18.00	12.25
No	2.34	0.70	1.64	46.94	45.80	45.44	38.85	19.51	10.76
Arrhythmia	1.233	0.424	1.320	0.409	1.414	-0.931	-1.549	-0.801	-0.041
Yes	3.25	0.80	2.45	47.70	48.40	44.30	36.64	18.80	10.78
No	2.17	0.68	1.50	46.95	45.25	45.67	39.20	19.59	10.81
Cancer	0.290	0.588	0.137	0.223	1.008	-0.013	1.542	-0.827	2.016^*^
Yes	2.80	1.00	1.80	47.80	49.80	45.40	43.40	18.00	13.34
No	2.32	0.69	1.64	47.04	45.58	45.46	38.62	19.52	10.71
Other	1.994^*^	2.124^*^	1.615	0.756	-2.936^**^	0.729	0.387	1.556	-0.303
Yes	3.84	1.32	2.53	48.26	40.19	46.39	39.37	20.79	10.58
No	2.08	0.59	1.49	46.86	46.70	45.30	38.71	19.24	10.85
Times of weekly hemodialysis	0.636	1.703	0.119	2.628^*^	-0.578	0.141	0.767	1.202	-0.185
2	3.13	1.38	1.75	53.63	43.92	45.75	40.60	21.13	10.63
3	2.29	0.65	1.64	46.63	45.86	45.44	38.69	19.36	10.82
Vintage of hemodialysis^a^	1.657	0.831	1.885	2.761	3.783^*^	1.133	0.216	0.462	0.616
Less than 5 years	1.73	0.56	1.17	47.34	43.95	45.64	39.18	19.75	10.76
6–10 years	3.00	0.86	2.14	44.92	49.08	46.31	38.74	18.94	11.21
> 10years	2.70	0.76	1.94	49.00	45.19	44.18	38.21	19.54	10.45
Post Hoc					2 > 1				
Primary reasons for choosing the current dialysis method^a^	0.431	0.375	0.403	0.285	0.093	1.115	0.139	0.400	0.642
Doctor’s recommendation	2.40	0.72	1.67	46.83	45.74	44.87	38.95	19.66	10.62
Personal preference	2.34	0.68	1.66	47.36	45.61	46.54	38.45	19.05	11.20
Family’s expectations	0.00	0.00	0.00	50.50	48.50	46.00	40.50	20.50	10.00
Post Hoc									
(Formerly) Engaged in medical-related work^t^	1.353	1.067	1.390	-0.604	-0.076	-0.642	-0.131	-0.155	-0.563
Yes	4.00	1.13	2.88	44.63	45.50	44.13	38.50	19.25	10.25
No	2.23	0.67	1.56	47.23	45.76	45.54	38.83	19.48	10.85
Feeling uncertain about the current dialysis decision^t^	-2.462^*^	-1.049	-2.983^**^	0.696	-0.742	-0.426	-0.557	-0.424	-2.160^*^
Yes	0.80	0.40	0.40	48.33	44.08	44.83	37.57	19.05	9.31
No	2.54	0.74	1.81	46.90	45.96	45.54	38.97	19.52	11.01
Have already signed a Do Not Resuscitate (DNR) order or an Advance Directive for Palliative and Life-Sustaining Treatment^t^	1.189	1.371	1.313	2.526^*^	-0.944	0.030	-0.573	1.561	-1.978
Yes	3.47	1.06	2.41	51.24	43.78	45.50	37.92	20.88	9.53
No	2.17	0.64	1.53	46.44	46.04	45.45	38.94	19.25	11.00
Healthcare professionals have discussed the prognosis and estimated survival time of dialysis with you and your family^t^	1.526	1.571	1.482	1.875	0.463	-0.091	-0.206	1.873	0.224
Yes	3.79	1.16	2.63	50.00	46.64	45.34	38.51	21.05	10.95
No	2.09	0.62	1.47	46.56	45.58	45.48	38.86	19.19	10.79
Healthcare professionals have discussed medical decisions in case the condition worsens and becomes life-threatening with you or your family^t^	1.011	1.404	0.902	3.167^**^	-0.748	0.569	0.978	1.399	0.213
Yes	3.33	1.06	2.28	52.06	44.23	46.45	40.27	20.44	10.94
No	2.18	0.64	1.54	46.26	45.98	45.30	38.57	19.31	10.79
Healthcare professionals have provided you or your family with information about Advance Care Planning (ACP) and Advance Directive (AD)^t^	2.499^*^	2.595^*^	1.538	1.143	-0.134	0.779	-0.524	0.413	-1.271
Yes	6.20	2.00	4.20	50.80	45.20	47.52	36.20	20.20	9.20
No	2.19	0.65	1.54	46.92	45.76	45.37	38.91	19.44	10.87
Have previously discussed medical decisions in case the condition worsens and becomes life-threatening with your family^t^	3.794^***^	3.541^**^	3.647^**^	3.585^***^	-0.594	2.318^*^	2.726^**^	4.118^***^	-1.297
Yes	4.28	1.28	3.00	50.43	45.02	47.27	41.19	21.53	10.32
No	1.47	0.44	1.03	45.56	46.06	44.64	37.73	18.54	11.03

Post Hoc: scheff

^*^P < 0.05

^**^P < 0.01

^***^P < 0.001

^t^t-test

^a^ANOVA

In terms of attitude, age differences emerged in ACP and AD goals (*p* < 0.05), PSDA (*p* < 0.05), and ACP importance (*p* < 0.05), although post hoc tests revealed no intergroup variance. Higher education correlated with more favorable attitudes toward ACP and AD goals (*p* < 0.01) and PSDA. Patients on dialysis for 6–10 years held stronger attitudes regarding ACP and AD barriers (*p* < 0.05) compared to those treated for under 5 years. Individuals who signed DNR or ACP documents exhibited higher attitudes toward ACP and AD goals (*p* < 0.05). Participants engaging in discussions about life-threatening decisions with healthcare providers or family displayed higher attitudes toward ACP and AD goals (*p* < 0.01) compared to those who hadn’t discussed. Furthermore, those discussing these decisions with family showed higher attitudes toward ACP and AD goals (*p* < 0.001), PSDA (*p* < 0.05), and ACP importance (*p* < 0.01) than non-discussants.

Regarding the intention to sign, females (*p* < 0.001), individuals with cardiovascular diseases (*p* < 0.05), and those discussing life-threatening medical decisions with their families (*p* < 0.001) exhibited a stronger inclination. Concerning the intention for life-sustaining treatment, individuals with sufficient savings or retirement funds displayed notably higher levels compared to those relying on family and friends (*p* < 0.05). Additionally, participants without uncertainty about their current dialysis decisions had significantly higher levels compared to those with uncertainty (*p* < 0.05).

### Associations between other related questions and knowledge, attitudes, and intentions

In Table 3, we summarize associations between related questions and knowledge, attitudes, and intentions. Patients generally believe that ACP should be initiated by themselves. Patients’ knowledge about ACP significantly surpassed that of nephrology physicians in overall knowledge (*p* < 0.001), ACP and AD knowledge (*p* < 0.01), and PSDA knowledge (*p* < 0.001). Patients without designating another healthcare proxy showed notably higher overall knowledge (*p* < 0.05) and PSDA knowledge (*p* < 0.05) compared to those designating someone else.

**Table 3:** Associations between other related questions and knowledge, attitudes, and intentions

Personal characteristics	All knowledge	Knowledge of ACP and AD	Knowledge of PDSA	Attitude of goal for ACP and AD	Attitude of barrier to ACP and AD	Attitude of PSDA	Importance of ACP	Intention to sign	Intention to life-support treatment
**“Most” want to discuss your end-of-life wishes with whom**	0.076	0.235	0.079	1.644	1.978	4.709^*^	2.717	4.079^*^	3.697^*^
None	2.13	0.69	1.44	44.87	45.63	42.38	35.44	16.81	9.98
Healthcare professionals	2.59	0.86	1.73	46.09	49.14	48.27	40.58	19.70	12.21
Family member	2.39	0.67	1.72	48.06	44.79	45.38	38.94	19.88	10.56
Post Hoc						2 > 1		3 > 1	
**Most” prefer to undergo Advance Care Planning (ACP) at which time**	1.278	1.735	0.953	1.091	1.785	4.214^**^	1.675	1.285	1.812
When health	2.46	0.74	1.72	47.51	43.84	44.38	38.79	19.87	10.84
When kidney function starts to decline	1.53	0.42	1.11	45.95	46.40	42.84	36.26	18.45	9.47
When kidney function deteriorates and requires dialysis	1.45	0.36	1.09	47.45	45.45	46.45	39.45	19.07	10.82
When other severe complications start to arise	3.71	1.21	2.50	48.46	47.86	49.07	41.29	20.53	11.63
When in a life-threatening situation	2.00	0.44	1.56	42.88	50.89	46.51	40.78	17.78	11.74
Post Hoc						4 > 1, 2			
**Believe that Advance Care Planning (ACP) should be initiated by whom “most”?**	8.660^***^	6.643^**^	8.973^***^	1.100	1.138	3.294^*^	2.437	2.390	1.266
Patient himself/herself	3.84	1.06	2.78	47.84	45.64	46.46	40.46	20.36	11.08
Nephrologist	1.19	0.33	0.87	46.98	45.14	44.34	37.65	18.72	10.50
Others	2.13	1.00	1.13	43.63	50.25	49.01	38.86	19.84	12.00
Post Hoc	**1 > 2**	**1 > 2**	**1 > 2**						
**Whom do you want to designate as your healthcare proxy?**^**t**^									
Spouse	0.317	-0.776	0.796	0.665	0.379	1.196	0.938	-1.119	0.698
Yes	2.43	0.63	1.81	47.46	46.01	46.02	39.31	19.11	10.97
No	2.23	0.79	1.44	46.58	45.39	44.74	38.17	19.91	10.61
Children	-1.122	-1.153	-1.031	-0.863	1.708	0.267	-0.774	1.215	-0.751
Yes	1.98	0.58	1.41	46.50	47.12	45.60	38.34	19.90	10.62
No	2.69	0.82	1.88	47.63	44.38	45.32	39.27	19.04	11.00
Siblings	1.863	1.315	2.019	3.175^**^	-2.253^*^	3.737^**^	2.864^**^	1.827	0.470
Yes	4.18	1.12	3.06	52.24	41.14	49.29	43.11	21.12	11.12
No	2.06	0.63	1.43	46.28	46.44	44.88	38.15	19.22	10.76
Friend	1.055	0.977	1.019	0.082	-1.203	9.775^***^	0.040	-0.340	0.584
Yes	5.00	1.50	3.50	47.50	38.00	52.50	39.00	18.50	12.00
No	2.30	0.69	1.61	47.06	45.86	45.35	38.80	19.48	10.79
Others	-2.165^*^	-0.812	-2.657^*^	-2.131^*^	0.086	-1.970	-2.637^**^	-2.066^*^	-0.297
Yes	1.00	0.47	0.53	43.27	45.93	42.60	34.53	17.47	10.60
No	2.52	0.73	1.79	47.57	45.71	45.83	39.37	19.73	10.84
**How much are you willing to pay out-of-pocket for participating in Advance Care Planning (ACP)? (US dollars)**	0.673	0.492	0.997	4.514^**^	1.715	4.123^**^	2.640^*^	1.248	2.283
33	2.14	0.69	1.45	45.82	45.38	44.15	37.88	19.18	10.31
66	3.78	1.00	2.78	53.44	44.44	49.00	42.10	21.78	11.33
99	2.92	0.92	2.00	51.15	46.28	50.38	42.46	20.92	12.69
165	5.00	1.50	3.50	49.50	32.00	52.00	45.00	20.87	9.00
197	3.00	1.00	2.00	35.00	57.00	49.00	37.00	18.00	14.69
263	4.00	0.00	4.00	58.00	36.17	46.00	47.92	22.00	10.50
Post Hoc									
**Believe that the “Advance Care Planning (ACP) consultation fees” should be paid by whom:**	4.684^**^	5.241^**^	3.830^**^	0.727	1.666	3.725^**^	1.407	3.947^**^	3.338^*^
Pay in full by oneself	4.18	1.36	2.82	48.00	41.82	49.18	38.45	21.00	8.73
Fully covered by National Health Insurance	1.98	0.56	1.42	46.61	45.56	43.72	38.04	18.74	10.34
Fully subsidized by the government	0.69	0.14	0.55	45.89	47.28	45.14	38.30	18.53	11.71
Fully funded by a charitable foundation	6.00	1.67	4.33	46.67	55.67	47.33	36.00	17.00	11.67
Shared equally between oneself and relevant parties	3.74	1.22	2.52	49.00	44.98	47.87	41.48	21.70	11.62
Post Hoc								5 > 2	

^*^P < 0.05

^**^P < 0.01

^***^P < 0.001

^t^t-test

Opinions on “ACP consultation fees” significantly varied in overall knowledge (*p* < 0.01), ACP and AD knowledge (*p* < 0.01), and PSDA knowledge (*p* < 0.01). Post hoc tests indicated that the willingness to pay fully or share costs equally with relevant parties was higher than full government subsidies, with no knowledge intergroup differences for PSDA. Patients most willing to discuss end-of-life topics with family showed more positive PSDA attitudes (*p* < 0.05) and different intention to continue LST with severe complications (*p* < 0.05). However, post hoc tests revealed no intergroup differences. Patients not designating another healthcare proxy had significantly higher intention to sign their intentions (*p* < 0.05) compared to those who designated someone else. Those favoring shared payment for ACP consultation fees exhibited significantly higher intention to sign their intentions (*p* < 0.05) than those favoring full National Health Insurance coverage. While intention to continue LST differed (*p* < 0.05), post hoc tests found no intergroup variance.

The intention to discuss end-of-life matters with healthcare providers significantly increased in patients with a positive PSDA attitude (*p* < 0.05) compared to those who preferred not discussing it. Preference for discussing such matters during severe complications was notably higher in patients with a positive PSDA attitude (*p* < 0.05) compared to discussing it when healthy or with decreased kidney function. Differences in opinions about initiating ACP were significant in terms of PSDA attitude (*p* < 0.05), but post hoc tests found no intergroup differences.

Patients designating siblings as healthcare proxies exhibited significantly higher scores in ACP and AD goals (*t* = 3.175, *p* < 0.01), PSDA (*p* < 0.01), and ACP importance (*p* < 0.01) compared to non-designators. However, they scored lower in ACP and AD barriers (*t* = -2.253, *p* < 0.05). Patients designating friends as healthcare proxies had significantly higher PSDA attitude scores (*p* < 0.001). Differences in money willingness for ACP consultations were significant in ACP and AD goals (*p* < 0.01), PSDA attitude (*p* < 0.01), and ACP importance (*p* < 0.05). However, post hoc tests revealed no intergroup differences. Differences in opinions about bearing “ACP consultation fees” were significant in terms of PSDA attitude (*p* < 0.05), but post hoc tests showed no intergroup variance.

The intention to discuss end-of-life matters with family was notably higher in individuals willing to sign their intentions (*p* < 0.05) than those not interested in discussing. Significant differences existed in the intention to continue LST (*p* < 0.05), but post hoc tests showed no intergroup variance. Patients abstaining from designating another healthcare proxy exhibited higher intention to sign their intentions (*p* < 0.05) compared to those designating someone else. Those believing in equally shared ACP consultation fees between themselves and others displayed higher intention to sign their intentions (*p* < 0.05) compared to those favoring full National Health Insurance coverage. Though intention to continue LST was significant (*p* < 0.05), post hoc tests found no intergroup differences.

## Discussion

This study constitutes the inaugural descriptive investigation simultaneously evaluating dialysis patients’ knowledge, attitudes, and intentions concerning ACP, AD, and PSDA. The demographic profile of our study cohort closely resembles that of a preceding study conducted in Taiwan [29], encompassing age, gender, dialysis duration, education, marital status, and religious affiliations. Unlike the aforementioned study [29], which primarily focused on ACP, our research expands its purview to encompass AD and PSDA. While their study pioneered the evaluation of all ACP components (ACSR-ACP), including awareness, contemplation, self-efficacy, and readiness, our investigation broadens patient autonomy from ACP to include AD and PSDA. Moreover, this study represents the inaugural attempt to explore knowledge and attitudes among dialysis patients within this specific domain. We are confident that our meticulously designed questionnaire comprehensively captures diverse aspects—knowledge, attitudes, and intentions—thus facilitating the identification of potential factors associated with ACP, AD, and PSDA.

In the previous study conducted in Taiwan [29], it was observed that approximately half of the participants lacked information about ACP. In our cohort, merely 10% of patients reported receiving ACP-related information from healthcare providers, resulting in a low DNR rate of 13.2% and only 14% engaging in discussions about critical life-threatening decisions. Furthermore, awareness rates for both ACP and AD, as well as PSDA, remained below 30%. Additionally, only 69.52% of participants expressed willingness to sign for ACP. However, patients exhibited positive attitudes (with standardized scores of 78.45 for ACP and AD objectives, 69.94 for PSDA, and 77.62 for ACP importance).

In discussions regarding ACP, nearly half (47.3%) believed that it should commence when an individual is healthy. Moreover, over half (51.9%) of the patients indicated a preference for nephrologists to primarily initiate ACP discussions. Furthermore, three-quarters of the patients could afford a self-paid consultation fee of 33 USD. Consequently, the primary limitation regarding ACP, AD, and PSDA pertains to the accessibility and comprehension of information. This observation aligns with another study [30] in Taiwan, which revealed that participants with access to palliative care information were 3.708 times more likely or willing to sign an AD. Collectively, these findings underscore the crucial role of healthcare professionals in initiating ACP at the earliest possible opportunity.

We recommend providing comprehensive information on ACP, AD, and PSDA to all dialysis patients. Commencing ACP early in their treatment trajectory is beneficial for dialysis patients and ideally should precede the onset of any clinical complications. This holds particular significance for dialysis patients, considering their relatively low five-year survival rate of 50%. As highlighted in a published study [31], there exist barriers to ACP among seriously ill Chinese patients and their families. However, in current clinical practice, logistical challenges, including workload constraints [32] and lack of feasible compensation, hinder the universal initiation of ACP for every patient.

Building on previous reports [4], we acknowledge that certain medical conditions prompt patients to sign AD, including anemia, advanced age, sepsis, frequent hospitalizations, and prolonged hemodialysis duration. In our study, we propose the possibility of establishing priority groups among patients. Offering information to these prioritized patients initially might potentially yield a higher success rate. Consequently, this information could be subsequently extended to the broader patient population. Based on our study findings, individuals aged 50 to 64 with at least a college education, unmarried status, and no history of diabetes might be prioritized for receiving this information.

Moreover, promoting discussions among patients and their families is crucial. Our study reveals that individuals who have previously engaged in medical decision discussions regarding deteriorating health conditions and life-threatening situations with family members demonstrated significantly higher overall knowledge (*p* < 0.001), ACP and AD knowledge (*p* < 0.01), and PSDA knowledge (*p* < 0.001) compared to those who had not engaged in such discussions with family members.

ACP and AD in the context of dialysis present notable distinctions compared to other medical scenarios. Firstly, nephrologists frequently interact with dialysis patients, often meeting them two to three times weekly during three-hour sessions. This regularity offers nephrologists ample opportunities to explore patients’ desires, understand their apprehensions, and evaluate their encounters. Secondly, individuals undergoing hemodialysis commonly grapple withCKD, signifying the presence of concurrent conditions. The persistent nature of their ailments fosters heightened awareness compared to those with acute illnesses. Thirdly, relatives accompanying dialysis patients during treatments actively partake in ACP discussions within the hemodialysis unit. Moreover, nephrological clinicians are often akin to extended family members [33], indicative of close relationships. In our study, we discovered that patients engaging in conversations with their families were more likely to possess enhanced knowledge, display favorable attitudes, and demonstrate increased readiness to sign AD.

This study has several notable limitations. Firstly, it is a single-center study, potentially limiting the generalizability of our findings to a broader population. Secondly, we did not thoroughly investigate the associations among knowledge, attitude, and intention in this study. There might be causal relationships among these factors, and further research is planned to address this aspect. If a positive correlation with causal effects is identified, the implementation of ACP for AD could potentially enhance knowledge and attitude among the patient population. Thirdly, the response rate was relatively low, at 50%, largely due to the advanced age of the participants in our institution. This may suggest that non-participants self-selected, potentially introducing selection bias into our study. This age-related factor might have influenced the study’s generalizability. Fourthly, our research exclusively focused on hemodialysis patients, omitting patients with ESKD undergoing peritoneal dialysis. We aim to explore the differences between hemodialysis and peritoneal dialysis in this context.

Despite these limitations, this study boasts several strengths. It represents the first comprehensive assessment of the status of ACP, AD, and the PSDA among hemodialysis patients. Additionally, this study utilizes newly developed and meticulously designed questionnaires with robust validation. These questionnaires cover various detailed aspects related to the subject matter. We believe that clinicians can utilize these questionnaires for further surveys involving patients undergoing hemodialysis.

## Conclusion

Hemodialysis patients demonstrated a significant knowledge deficit regarding ACP, AD, and the PSDA. Remarkably, a considerable number of these patients lacked sufficient information on these topics. Despite this, they showcased a positive attitude, with a substantial portion expressing readiness to sign AD. Nephrologists play a crucial role in initiating ACP discussions with patients right from the outset, which is imperative in this context.

### Supplementary Information

**Additional file 1: S Table 1.** List of experts participating in the content validity assessment. **S Table 2.** Operational Definition of Patient Basic Characteristics. **S Table 3.** Operational Definition of Other Related Questions. **S Table 4.** Detailed information of this questionnaire. **S Table 5.** Complete Questionnaire Content (translate into English). **S Table 6.** Reliability Analysis of the Knowledge, Attitudes, and Willingness Scale on Advance Care Planning (ACP) and Patient Autonomy Act among Hemodialysis Patients. **S Table 7.** Hemodialysis patients' knowledge, attitude, and intentions regarding ACP, AD and PSDA. **S Figure 1.** Research Framework.

## Data Availability

All data generated or analyzed during this study are included in this published article and its supplementary information files.
