# Supplementary material for: Exploring knowledge, attitude, and intention towards advance care planning, advance directive, and the patient self-determination act among hemodialysis patients
Source: BMC Palliat Care. 2023 Dec 14;22:201. doi: 10.1186/s12904-023-01321-2 (PMC10720199; doi:10.1186/s12904-023-01321-2)
Supplement: Supplementary file 1 — Additional file 1: S Table 1. List of experts participating in the content validity assessment. S Table 2. Operational Definition of Patient Basic Characteristics. S Table 3. Operational Definition of Other Related Questions. S Table 4. Detailed information of this questionnaire. S Table 5. Complete Questionnaire Content (translate into English). S Table 6. Reliability Analysis of the Knowledge, Attitudes, and Willingness Scale on Advance Care Planning (ACP) and Patient Autonomy Act among Hemodialysis Patients. S Table 7. Hemodialysis patients' knowledge, attitude, and intentions regarding ACP, AD and PSDA. S Figure 1. Research Framework. [file 12904_2023_1321_MOESM1_ESM.docx]

Supplementary data

S Table 1. List of experts participating in the content validity assessment

| No | Service organization | Job title / Position / Title |
| --- | --- | --- |
| 1 | Taichung veteran general hospital | Attending Physician in the Palliative Care Department of the Family Medicine Division |
| 2 | Taichung veteran general hospital | Deputy Head Nurse of the Hemodialysis Unit |
| 3 | Taichung veteran general hospital | Director of Palliative Care Unit |
| 4 | WelGong Memorial Hospital | Attending Physician in Nephrology Department and Director of Peritoneal Dialysis Unit |
| 5 | Taichung veteran general hospital | Director of Palliative Care Unit in the Family Medicine Department |
| 6 | Department of Healthcare Administration in Asia University | Associate Professor |
| 7 | Taichung veteran general hospital | Director of Nursing Department (Overseeing Palliative Care Unit) |
| 8 | Taichung veteran general hospital | Head of Nephrology Department |
| 9 | Ditmanson Medical Foundation Chia-Yi Christian Hospital | Director of Nursing Department (Overseeing Dialysis Services) |
| 10 | Taichung veteran general hospital | Head Nurse of Palliative Care Unit |
| 11 | Department of Healthcare Administration in Central Taiwan University of Science and Technology | Associate Professor |
| 12 | Taichung veteran general hospital | Former Director of Palliative Care Unit |

S Table 2. Operational Definition of Patient Basic Characteristics

| Independent variable | Operational definition | types |
| --- | --- | --- |
| Demographic | | |
| Gender | Male or female | Categorical |
| Age | Age while recruitment | Continuous |
| Level of education | "Below Junior High School," "Junior High School," "Senior High School / Vocational School," "Junior College," "University," "Graduate School and Above" | Categorical |
| marriage | "Married," "Single," "Divorced, Separated," "Widowed," "Other" | Categorical |
| Religious Belief | "No Religion," "Chinese Folk Religion," "Buddhism," "Taoism," "Catholicism," "Christianity," "Other" | Categorical |
| Residential Status | "Living Alone," "Living with Family," "Living with Others" | Categorical |
| Residential Status | "Work income is sufficient to support" "Savings or retirement are sufficient to support" "Depend on family or friends for support" "Depend on social assistance for support" | Categorical |
| Comorbidity | "Hypertension" ,"Cardiovascular disease" ,"Diabetes" ,"Heart failure" ,"Ischemic heart disease" ,"Dyslipidemia" ,"Peptic ulcer" ,"Stroke" ,"Arrhythmia", "Chronic Obstructive Pulmonary Disease (COPD)", "Cancer", "Other" | Categorical |
| Information of dialysis |  |  |
| Vintage of dialysis | Times in years | Continuous |
| Why did you choose current dialysis model | "Physician's Wishes," "Personal Wishes," "Family's Wishes" | Categorical |
| Do you regret the decision for dialysis? | Yes or no | Categorical |
| **Other associated experience** |  |  |
| Have you signed a Do-Not-Resuscitate (DNR) form or an Advance Care Planning (ACP) and Advance Directive (AD) form for palliative care and life-sustaining treatment decisions? | Yes or no | Categorical |
| Have you had discussions with a physician or healthcare personnel regarding the prognosis and estimated survival time for dialysis? | Yes or no | Categorical |
| Have healthcare providers discussed with you and your family about medical decisions for dialysis treatment during end-of-life care? | Yes or no | Categorical |
| Have physicians or healthcare providers provided information to you and your family about Advance Care Planning (ACP) and Advance Directive (AD) for medical decision-making? | Yes or no | Categorical |

S Table 3. Operational Definition of Other Related Questions

| Independent variable | Operational definition | types |
| --- | --- | --- |
| Who do you "most" want to discuss your end-of-life wishes with? | "No one", "Nephrologist", "Palliative care physician", "Other specialist physician", "Dialysis nurse", "Palliative care nurse", "Spouse", "Children", "Siblings", "Friends", "Other" | Categorical |
| When do you "most" prefer to have Advance Care Planning (ACP)? | "When in good health", "When discovering decreased kidney function", "When kidney function deteriorates to require dialysis", "When other severe complications begin to appear", "When in a life-threatening situation" | Categorical |
| Who do you think "most" should initiate the ACP process? | "The patient themselves," "Nephrologist," "Palliative care physician," "Other specialist physician," "Dialysis nurse," "Palliative care nurse," "Close family members," "Others" | Categorical |
| Whom would you like to designate as your appointed agent? | "Spouse," "Children," "Siblings," "Friends," "Lawyer," "Others" | Categorical |
| How much are you willing to self-fund for participating in Advance Care Planning (ACP)? | 「33」、「66」、「99」、「132」、「165」、「197」、「263、「328」  US dollars | Categorical |
| Who do you think should bear the cost of "Advance Care Planning fees"? | "Self-pay in full," "Fully covered by National Health Insurance," "Fully subsidized by the government," "Fully covered by a charitable foundation," "Shared equally between myself and other relevant parties" | Categorical |

S Table 4. Detailed information of this questionnaire

| The first section  (baseline data) | For ths first section, we inclued the following variables: demographic variables (gender, age, education level, marital status, religious beliefs, living situation, economic status, other significant illnesses), dialysis treatment details (duration of undergoing dialysis treatment, reasons for choosing the current dialysis method, and any regrets regarding the dialysis decision), experiences related to ACP (whether the participant has previously signed a Do-Not-Resuscitate (DNR) order or an Advance Directive for Palliative and Life-Sustaining Treatment, whether a physician or healthcare provider has discussed prognosis and anticipated survival time related to dialysis, medical decisions regarding dialysis treatment during end-of-life, and information about ACP and AD. |
| --- | --- |
| The second section (knowledge) | For ths second section (knowledge), we employs a binary scoring method with a total of 10 questions. Participants are required to mark 'Yes' based on their own perceptions." |
| The third section (altitute) | In the attitude section, there are four dimensions, including the goals of Advance Care Planning (ACP) and Advance Directives (AD), obstacles to ACP and AD, and patients' autonomy rights. This section comprises a total of 41 items, assessed using a Likert Scale five-point system. Respondents are to indicate their agreement based on their experiences and perceptions, with 'Strongly Agree' being assigned 5 points, 'Agree' - 4 points, 'Neutral' - 3 points, 'Disagree' - 2 points, and 'Strongly Disagree' - 1 point. The total score ranges from 41 to 205, with higher scores indicating a more positive attitude. The other part involves importance and consists of 10 items, also assessed using a Likert Scale five-point system. Participants are asked to indicate the importance according to their feelings, with 'Very Important' assigned 5 points, 'Important' - 4 points, 'Neutral' - 3 points, 'Not Important' - 2 points, and 'Not at All Important' - 1 point. The total score ranges from 10 to 50, with higher scores indicating greater importance." |
| The fourth section (willingness) | In the willingness section (fourth section), there are two dimensions, including willingness to participate and willingness to sign, as well as willingness to maintain life-sustaining treatment. This section consists of a total of 12 items, assessed using a Likert Scale five-point system. Respondents are asked to indicate their willingness, with 'Very Willing' assigned 4 points, 'Willing' - 3 points, 'Unwilling' - 2 points, and 'Very Unwilling' - 1 point. The total score ranges from 12 to 48, with higher scores indicating a higher level of willingness. |
| The fifth section | In the fifth section, This includes questions such as: "Who would you most like to discuss your end-of-life wishes with?" "When would you prefer to engage in Advance Care Planning (ACP)?" "In your opinion, who should initiate Advance Care Planning (ACP)?" "Whom would you like to designate as your surrogate decision-maker?" "How much would you be willing to pay out of pocket to participate in Advance Care Planning (ACP)?" "In your view, who should be responsible for covering the cost of Advance Care Planning (ACP)?" |

S Table 5. Complete Questionnaire Content (translate into English)

**Questionnaire on Knowledge, Attitudes, and Preferences of Hemodialysis Patients towards Advance Care Planning and Patient Autonomy Rights**

| "Dear Kidney Patient,  Greetings! We are a research team from the Nephrology Department of Taichung Veterans General Hospital and the Health Care Administration Department of Chung-Tai University of Science and Technology. We are currently conducting a study titled 'Knowledge, Attitudes, and Preferences of End-Stage Renal Disease Patients towards Advance Care Planning and Patient Autonomy Rights.' Your valuable input will help us understand the intentions of end-stage renal disease patients and their families regarding advance care planning and patient autonomy rights. This study aims to provide insights for future reference in matters related to advance care planning and patient autonomy rights.  (Before completing the questionnaire, please read the provided glossary for detailed explanations.)  This questionnaire is designed to be completed anonymously. All collected data will be used solely for academic research and analysis purposes. Your responses will be kept confidential and will not be disclosed to external parties. Your participation is highly appreciated.  Thank you!"  Taichung Veterans General Hospital  Department of Nephrology  Director: Dr. Chen Cheng-Hsu  Chung-Tai University of Science and Technology  Department of Health Care Administration  Associate Professor: Dr. Yeh Te-Feng  Researcher:  Yang Jia-Yi  Contact Information:  Email: ppkk06260702@gmail.com  Phone: 0933-292-140 |
| --- |

Glossary

- Life-sustaining treatment: Refers to any necessary medical interventions, such as cardiopulmonary resuscitation (CPR), mechanical life-support systems, blood products, specialized treatments for specific diseases, and administration of antibiotics during severe infections, which have the potential to prolong a patient's life.
- Artificial nutrition and hydration: Refers to the provision of food and fluids through tubes or other invasive methods.
- Advance Care Planning (ACP): Refers to the communication process between patients, healthcare providers, family members, or other relevant individuals. It involves discussing appropriate care options for a patient when they are in specific clinical conditions, unconscious, or unable to express their wishes clearly. This includes decisions about life-sustaining treatment and artificial nutrition and hydration that the patient can accept or refuse.
- Advance Directive (AD): Refers to a pre-established written statement indicating a person's preferences for receiving or refusing life-sustaining treatment, artificial nutrition and hydration, or other relevant decisions related to medical care, end-of-life wishes, etc., for specific clinical conditions.
- Medical Surrogate Decision-Maker: Refers to a person designated in writing by an individual to express their wishes when the individual is unconscious or unable to communicate their wishes.
- Specific Clinical Conditions: Refers to meeting one of the following clinical conditions: 1) Terminal illness; 2) Irreversible coma; 3) Permanent vegetative state; 4) Profound dementia; 5) Other diseases or conditions announced by the central competent authority, characterized by unbearable suffering or lack of available treatment options according to the prevailing medical standards.
- End of life: Refers to the point when various medical interventions can no longer cure or control the progression of a disease.

Session 1. Background Information

1. Gender：

□male 　□female

1. Age：
2. Educational level：
3. □ Elementary school or below □ Junior high school □ High school/vocational school □ Junior college □ University □ Postgraduate"
4. Marriage：
5. □ Married □ Single □ Divorced, Separated □ Widowed □ Other宗教信仰：
6. Living Situation:

□ Living alone □ Living with family □ Living with others"

1. Economic Situation:

□ Sufficient income from work □ Sufficient savings or retirement funds □ Dependence on family and friends for support □ Dependence on social assistance for support"

1. Do you currently have any other significant illnesses? (Select all that apply)

□ Hypertension □ Cardiovascular disease □ Diabetes □ Heart failure □ Ischemic heart disease □ Blood lipid abnormalities

□ Peptic ulcer □ Stroke □ Arrhythmia □ Chronic obstructive pulmonary disease □ Cancer □ Other: ___________

1. The dialysis method you currently use:

□ Peritoneal Dialysis (□ APD □ CAPD □ APD+CAPD)

□ Hemodialysis (□ 2 times a week □ 3 times a week)

□ Mixed Peritoneal and Hemodialysis

1. Duration of receiving dialysis treatment: Since the year ______ of the Republic of China
2. What is the primary reason for choosing your current dialysis method?

□ Physician's recommendation □ Personal preference □ Family's expectation

1. Have you (ever) worked in a medical-related occupation?

□ Yes □ No

1. Do you have doubts about your current dialysis decision?□ Yes □ No
2. Have you signed a Do-Not-Resuscitate (DNR) order or an Advance Directive for Palliative and Life-Sustaining Treatment?

□ Yes □ No

1. Have healthcare professionals discussed the prognosis and expected survival time of dialysis with you and your family?

□ Yes □ No

1. Have healthcare professionals discussed medical decisions in case your condition worsens and becomes life-threatening with you or your family?

□ Yes □ No

1. Have healthcare professionals provided you or your family with information about Advance Care Planning (ACP) and Advance Directives (AD)?

□ Yes □ No

1. Have you ever discussed medical decisions in case your condition worsens and becomes life-threatening with your family?

□ Yes □ No"

Session 2、Knowledge of Advance Care Planning (ACP) and Patient Autonomy Rights

| "Please provide your responses to the following items regarding your 'Knowledge of Advance Care Planning (ACP) and Patient Autonomy Rights.' Please select the appropriate option that best matches your level of understanding based on the item descriptions (please place a X inside the □ box that you find suitable)." | Yes | No |
| --- | --- | --- |
| 1. Have you heard of Advance Care Planning (ACP) before today | □ | □ |
| 1. Have you heard of Advance Directives (AD) before today? | □ | □ |
| 1. Did you know that you can designate a medical surrogate decision-maker to express your medical wishes when you are unconscious or unable to communicate your preferences? | □ | □ |
| 1. Are you aware that Taiwan began implementing the Patient Autonomy Act in 2019 (Republic of China year 108)? | □ | □ |
| 1. Are you aware that under the Patient Autonomy Act, patients can sign an Advance Directive (AD) to decide in advance the treatment they wish to receive or refuse at the end of life? | □ | □ |
| 1. Are you aware that under the Patient Autonomy Act, healthcare providers can provide treatment according to a patient's Advance Directive if the patient is unable to make decisions themselves? | □ | □ |
| 1. Are you aware that under the Patient Autonomy Act, patients can appoint a medical surrogate decision-maker to make treatment decisions on their behalf when they cannot make decisions themselves? | □ | □ |
| 1. Are you aware that even after signing an Advance Directive (AD), you can still change your initial decision? | □ | □ |
| 1. Are you aware that you can have more than one medical surrogate decision-maker?" | □ | □ |
| "Please provide your responses to the following items regarding your 'Knowledge of Advance Care Planning (ACP) and Patient Autonomy Rights.' Please select the appropriate option that best matches your level of understanding based on the item descriptions (please place a X nside the □ box that you find suitable)." | Yes | No |
| 1. Are you aware that after signing an Advance Directive (AD), it needs to be recorded on the National Health Insurance IC Card to become effective?" | □ | □ |

Session 3. Attitudes towards Advance Care Planning (ACP) and Patient Autonomy Rights

| Please provide your responses to the following items regarding your 'Knowledge of Advance Care Planning (ACP) and Patient Autonomy Rights.' Please choose the appropriate option that best matches your level of understanding based on the item descriptions (please place a X inside the □ box that you find suitable | Strongly agree | Agree | No opinion | Disagree | Strongly disagree |
| --- | --- | --- | --- | --- | --- |
| 1. Advance Care Planning (ACP) can help you understand the various available care options at the end of life, along with their benefits and risks. | □ | □ | □ | □ | □ |
| 1. ACP can enhance the understanding of your values and preferences in end-of-life care for both your family and healthcare team, leading to consensus building. | □ | □ | □ | □ | □ |
| 1. ACP can reduce the likelihood of receiving ineffective medical treatment at the end of life. | □ | □ | □ | □ | □ |
| 1. ACP gives you more confidence in facing the worst outcomes of your illness, including death. | □ | □ | □ | □ | □ |
| 1. ACP can ensure your quality of life at the end of life. | □ | □ | □ | □ | □ |
| 1. ACP can lessen the psychological burden on your family when making medical decisions on your behalf. | □ | □ | □ | □ | □ |
| 1. ACP should involve regular discussions, allowing you to review the progress of various relevant medical interventions and whether your preferences need to be updated. | □ | □ | □ | □ | □ |
| 1. Given the higher risk of death in end-stage kidney disease (uremia) compared to other illnesses, do you think participating in ACP is necessary? | □ | □ | □ | □ | □ |
| 1. Do you believe that end-stage kidney disease (uremia) patients should begin discussing Advance Care Planning (ACP) in the early stages of dialysis?" | □ | □ | □ | □ | □ |
| 1. Advance Directives (AD) allow you to make medical decisions for yourself even when you lose decision-making capacity. | □ | □ | □ | □ | □ |
| 1. Implementing Advance Directives (AD) can reduce unnecessary suffering at the end of life. | □ | □ | □ | □ | □ |
| 1. Advance Directives (AD) enable you to make end-of-life care decisions based on your values and life goals, contributing to a peaceful end of life. | □ | □ | □ | □ | □ |
| 1. You believe that the appointed healthcare proxy will represent your medical decisions when you're unconscious or unable to express your wishes. | □ | □ | □ | □ | □ |
| 1. You are not yet ready to discuss Advance Care Planning (ACP). | □ | □ | □ | □ | □ |
| 1. Discussing Advance Care Planning (ACP) inevitably involves the topic of death, which may make life seem hopeless to you. | □ | □ | □ | □ | □ |
| 1. You think that doctors or nurses don't have sufficient education to discuss Advance Care Planning (ACP) with you and your family. | □ | □ | □ | □ | □ |
| 1. You believe that doctors or nurses don't have enough time to discuss Advance Care Planning (ACP) with you and your family. | □ | □ | □ | □ | □ |
| 1. The process of signing an Advance Directive (AD) involves contemplating death or incapacity, which can be unpleasant. | □ | □ | □ | □ | □ |
| 1. Signing an Advance Directive (AD) requires a significant amount of time. | □ | □ | □ | □ | □ |
| 1. If you sign an Advance Directive (AD), you worry about being abandoned by medical treatment. | □ | □ | □ | □ | □ |
| 1. Signing an Advance Directive (AD) is unnecessary as you trust your loved ones to make the right decisions on your behalf. | □ | □ | □ | □ | □ |
| 1. You think that even if you sign an Advance Directive (AD), you can't be sure that the medical team will follow your wishes when needed. | □ | □ | □ | □ | □ |
| 1. You're concerned that a signed Advance Directive (AD) may not cover all future medical decisions. | □ | □ | □ | □ | □ |
| 1. Signing an Advance Directive (AD) makes you fear a reduction in medical care by the healthcare team at the end of your life. | □ | □ | □ | □ | □ |
| 1. You have signed a Do Not Resuscitate (DNR) order or an Advance Directives for Hospice and Palliative Care, so you don't need to sign an Advance Directive (AD) again. | □ | □ | □ | □ | □ |
| 1. For you, appointing a healthcare proxy is unnecessary because you don't want to burden your family emotionally. | □ | □ | □ | □ | □ |
| 1. You don't believe that your current dialysis endangers your life, so you've never considered the issue of death. | □ | □ | □ | □ | □ |
| 1. You believe that for end-stage kidney disease (uremia) patients, extending life-sustaining treatment is more important than discontinuing life-sustaining treatment. | □ | □ | □ | □ | □ |
| 1. The Patient Autonomy Act only allows patients to exercise their right to refuse medical treatment, which can lead to natural death, not euthanasia. | □ | □ | □ | □ | □ |
| 1. Patients have the right to be informed about their condition, treatment options, potential outcomes, and risks, and the right to choose and decide about the treatment options provided by doctors, without interference from others. | □ | □ | □ | □ | □ |
| 1. During patient visits, the healthcare team should inform the patient or, if the patient does not object, the family or relevant individuals, about the patient's condition, treatment plan, procedures, medications, prognosis, and possible adverse reactions at an appropriate time and in a suitable manner. | □ | □ | □ | □ | □ |
| 1. When engaging in Advance Care Planning (ACP), the patient, at least one immediate family member within the second degree of kinship, and the healthcare proxy must all participate. | □ | □ | □ | □ | □ |
| 1. Signing an Advance Directive (AD) requires prior Advance Care Planning (ACP) with the healthcare team, institutional endorsement, notarization by a notary public, or witnessing by two or more individuals, and it must be documented on the National Health Insurance (NHI) IC card to be effective. | □ | □ | □ | □ | □ |
| 1. Even after signing an Advance Directive, you can still withdraw or modify it at any time in writing. | □ | □ | □ | □ | □ |
| 1. If the patient's most recent medical decision is to receive life-sustaining treatment or artificial nutrition and hydration, doctors should immediately execute the patient's wishes. | □ | □ | □ | □ | □ |
| 1. If the patient's most recent medical decision is to refuse life-sustaining treatment or artificial nutrition and hydration, doctors should still follow the original Advance Directive until the process of updating the IC card is completed. | □ | □ | □ | □ | □ |
| 1. The appointed healthcare proxy must be at least twenty years old and possess full legal capacity, and their appointment must be made in writing. | □ | □ | □ | □ | □ |
| 1. The healthcare proxy, when the patient is unconscious or unable to express their wishes, has the authority to represent the patient's medical decisions, including receiving information from the healthcare team, signing consent forms, and expressing the patient's medical wishes according to the content of the patient's Advance Directives. | □ | □ | □ | □ | □ |
| 1. If there are multiple healthcare proxies appointed, each of them can independently execute the patient's Advance Directives, without requiring all of them to be present. | □ | □ | □ | □ | □ |
| 1. If a patient meets any of the clinical conditions such as end-stage disease, irreversible coma, permanent vegetative state, severe dementia, and others, and has Advance Directives, the healthcare team can use the patient's Advance Directives to terminate, withdraw, or withhold all or part of life-sustaining treatment or artificial nutrition and hydration." | □ | □ | □ | □ | □ |
| 1. The healthcare team has the right, based on their expertise or discretion, to not carry out the patient's Advance Directives. | □ | □ | □ | □ | □ |

Session 4. The Importance of Advance Care Planning (ACP) for End-Stage Kidney (Uremia) Patients

| Please select the appropriate option (place a X inside the box) based on your perception of the importance of the following items regarding 'The Importance of Advance Care Planning (ACP) and Patient Autonomy Act | Very important | Important | Neutral | Not important | Very umimportant |
| --- | --- | --- | --- | --- | --- |
| 1. Enhancing patients and families' understanding of the illness, including prognosis, treatment options (dialysis treatment, conservative management, and palliative care services), and potential outcomes of these treatment choices. | □ | □ | □ | □ | □ |
| 1. Guiding patients in establishing care goals, including in scenarios of losing decision-making capacity and during end-of-life situations. | □ | □ | □ | □ | □ |
| 1. Develop care plans in alignment with patients' goals, incorporating their preferences for situations of loss of decision-making capacity, meeting specific clinical conditions, and end-of-life care. | □ | □ | □ | □ | □ |
| 1. Ensure that healthcare professionals make medical decisions in accordance with the patient's Advance Directive (AD) wishes. | □ | □ | □ | □ | □ |
| 1. Assist in maintaining the quality and dignity of life for patients, promoting emotional tranquility. | □ | □ | □ | □ | □ |
| 1. Strengthen opportunities for patients and their loved ones to discuss the patient's future care preferences. | □ | □ | □ | □ | □ |
| 1. Aid in alleviating the emotional burden experienced by patients and their loved ones during medical decision-making. | □ | □ | □ | □ | □ |
| 1. Assist patients in designating healthcare proxy agents for future care decisions. | □ | □ | □ | □ | □ |
| 1. Support healthcare proxy agents in understanding their roles and responsibilities in the patient's future medical decisions. | □ | □ | □ | □ | □ |
| 1. Facilitate mutual understanding among patients, healthcare proxy agents, and healthcare professionals regarding the patient's values and wishes. | □ | □ | □ | □ | □ |

Session 5. Willingness towards Advance Care Planning (ACP) and Patient Autonomy Rights

| Please choose the appropriate option (place a X □ inside the box) based on your willingness, considering the descriptions provided, regarding "Willingness towards Advance Care Planning (ACP) and Patient Autonomy Act": | Very willing | Willing | Unwilling | Very unwilling |
| --- | --- | --- | --- | --- |
| 1. Are you willing to participate in Advance Care Planning (ACP)? | □ | □ | □ | □ |
| 1. Are you willing to sign an "Advance Directive" (AD)? | □ | □ | □ | □ |
| 1. Are you willing to encourage your family members to participate in Advance Care Planning (ACP)? | □ | □ | □ | □ |
| 1. Are you willing to encourage your family members to sign an "Advance Directive" (AD)? | □ | □ | □ | □ |
| 1. Are you willing to sign a "Do Not Resuscitate (DNR) Order or Advance Care Planning and Life-sustaining Treatment Decision Directive"? | □ | □ | □ | □ |
| 1. Are you willing to sign an "Advance Medical Proxy Appointment Directive" to designate a medical proxy? | □ | □ | □ | □ |
| 1. Are you willing, under the recommendation of a physician, to reduce or discontinue dialysis during end-of-life or specific clinical conditions? | □ | □ | □ | □ |
| 1. If diagnosed with specific clinical conditions, would you be willing to accept enteral feeding through a "nasogastric tube"? | □ | □ | □ | □ |
| 1. If diagnosed with specific clinical conditions, would you be willing to accept treatment involving "tracheal intubation"? | □ | □ | □ | □ |
| 1. If diagnosed with specific clinical conditions, would you be willing to accept "cardiopulmonary resuscitation" (CPR) measures? | □ | □ | □ | □ |
| 1. If diagnosed with specific clinical conditions, would you be willing to accept "emergency medication administration" measures? | □ | □ | □ | □ |
| 1. If diagnosed with specific clinical conditions, would you consider stopping or reducing dialysis? | □ | □ | □ | □ |

**Other Related Questions about Advance Care Planning (ACP) and Patient Autonomy Law**

1. Who would you "most" like to discuss your end-of-life wishes with? (Choose one)

□ Don't want to discuss with anyone □ Nephrologist □ Palliative care physician □ Other specialist physician□ Dialysis nurse □ Palliative care nurse □ Spouse □ Children □ Siblings □ Friends

□ Other_____________

1. When would you "most" prefer to undergo Advance Care Planning (ACP)? (Choose one)

□ When I am healthy □ When I notice a decline in kidney function □ When kidney function worsens and requires dialysis □ When other serious complications arise □ When in a life-threatening situation

1. Who do you think should initiate Advance Care Planning (ACP) "most"?

□ The patient themselves □ Nephrologist □ Palliative care physician □ Other specialist physician□ Dialysis nurse □ Palliative care nurse □ Close family member □ Other_____________

1. Whom would you like to designate as your appointed agent? (Multiple choices allowed)

□ Spouse □ Children □ Siblings □ Friends □ Lawyer □ Other_____________

1. How much are you willing to pay out of pocket to participate in Advance Care Planning (ACP) consultation? (One-time) (US dollars)

□ $33 □ $66 □ $99 □ $133 □ $167 □ $197 □ $263 □ $333

1. Who do you think should bear the cost of "Advance Care Planning consultation fees"?

□ Myself, fully □ National Health Insurance, fully □ Government, fully □ Charitable Foundation, fully □ Split between myself and relevant organizations

S Table 6. Reliability Analysis of the Knowledge, Attitudes, and Willingness Scale on Advance Care Planning (ACP) and Patient Autonomy Act among Hemodialysis Patients.

|  | **Number of questions** | **Pre-rest(α)** | **Formal test(α)** |
| --- | --- | --- | --- |
| **Knowledge (KR20)** | 10 | 0.916 | 0.956 |
| **Altitude** |  |  |  |
| Goal of ACP and AD | 13 | 1.000 | 0.983 |
| Barrier of ACP and AD | 15 | 0.983 | 0.916 |
| patient right to autonomy act | 14 | 0.988 | 0911 |
| Importance | 10 | 0.916 | 0.985 |
| **Willingness** |  |  |  |
| Willingness to Agree | 7 | 0.976 | 0.965 |
| Willingness for Life Support Treatment | 5 | 0.950 | 0.843 |

Formal test: n=129

Pre-test: n=33

S Table 7. Hemodialysis patients' knowledge, attitude, and intentions regarding ACP, AD and PSDA

| Variable | Score | （%） | Mean | SD |
| --- | --- | --- | --- | --- |
| **All knowledge (0-10)** | **23.4** |  | **2.34** | **3.59** |
| ACP and AD (0-3) | 23.3 |  | 0.70 | 1.17 |
| Have you heard of Advance Care Planning (ACP) before today? |  | 22.0% |  |  |
| Have you heard of Advance Directives (AD) before today? |  | 22.0% |  |  |
| Before today, were you aware that you can designate a healthcare proxy to express your medical wishes on your behalf in the event of unconsciousness or inability to clearly communicate your preferences? |  | 26.0% |  |  |
| **PSDA (0-7)** | **23.4** |  | **1.64** | **2.60** |
| Did you know that Taiwan began implementing the Patient Autonomy Act in the year 2019 (Republic of China 108)? |  | 22.5% |  |  |
| Are you aware that under the Patient Autonomy Act, patients can sign Advance Directives (AD), which allow them to make advance decisions about the medical treatments they wish to receive or refuse at the end of life? |  | 27.9% |  |  |
| Are you aware that under the Patient Autonomy Act, healthcare providers can follow the treatment preferences outlined in a patient's Advance Directives when the patient is unable to make decisions for themselves? |  | 26.4% |  |  |
| Are you aware that under the Patient Autonomy Act, patients can designate a healthcare proxy who can make treatment decisions on their behalf when they are unable to make decisions for themselves? |  | 26.4% |  |  |
| Did you know that even after signing an Advance Directive (AD), you can still change your initial decision? |  | 22.5% |  |  |
| Are you aware that you can have more than one healthcare prox |  | 18.6% |  |  |
| Are you aware that after signing an Advance Directive (AD), it needs to be noted on your National Health Insurance (NHI) IC card to become effective? |  | 20.2% |  |  |
| **Attitude** | **Score** | **Mean** | | **SD** |
| Goal of ACP and AD (13-65) | 84.97 | 50.98 | | 8.00 |
| Advance Care Planning (ACP) can help you understand the various available care options and their benefits and risks during the end of life. |  | 3.95 | | 0.64 |
| ACP can enhance your family and medical team's understanding of your values and preferences for end-of-life care, leading to consensus building. |  | 3.94 | | 0.62 |
| ACP can reduce the likelihood of ineffective medical interventions during the end of life. |  | 3.93 | | 0.64 |
| ACP empowers you to face the worst outcomes of illness, including death, with increased confidence. |  | 3.96 | | 0.67 |
| ACP ensures your quality of life during the end of life. |  | 3.94 | | 0.66 |
| ACP can alleviate the psychological burden on your family when making medical decisions on your behalf. |  | 3.95 | | 0.69 |
| ACP discussions should be conducted periodically, allowing you to review the progress of various relevant medical interventions and whether your preferences have changed. |  | 3.89 | | 0.71 |
| Considering the higher risk of mortality in end-stage kidney disease (uremia) compared to other illnesses, do you think participating in ACP is necessary? |  | 3.92 | | 0.67 |
| Do you believe that patients with end-stage kidney disease (uremia) should initiate discussions about ACP early in the dialysis process? |  | 3.77 | | 0.79 |
| Advance Directives (AD) enable you to make decisions about your medical care even when you've lost decision-making capacity. |  | 3.93 | | 0.64 |
| Implementing AD can reduce unnecessary suffering during the end of life. |  | 3.96 | | 0.68 |
| AD involves making end-of-life care decisions based on your values and life goals, contributing to a peaceful passing. |  | 3.92 | | 0.69 |
| Do you have faith that your healthcare proxy will advocate for your medical decisions in accordance with your wishes when you're unconscious or unable to communicate your preferences clearly? |  | 3.91 | | 0.68 |
| **Barriers of ACP and AD (15-75)** | **52.29** | **41.83** | | **9.35** |
| You are not yet ready to engage in discussions about Advance Care Planning (ACP). |  | 3.02 | | 0.96 |
| Discussing Advance Care Planning (ACP) inevitably involves conversations about death, which can make you feel hopeless about life. |  | 2.68 | | 0.96 |
| You believe that physicians or healthcare professionals do not have sufficient education and training to discuss Advance Care Planning (ACP) with you and your family. |  | 2.78 | | 0.81 |
| You believe that physicians or healthcare professionals do not have enough time to discuss Advance Care Planning (ACP) with you and your family. |  | 2.91 | | 0.77 |
| The process of signing an Advance Directive (AD) requires contemplating death or incapacity, which can be unpleasant. |  | 2.69 | | 0.93 |
| Signing an Advance Directive (AD) requires a significant amount of time. |  | 2.84 | | 0.85 |
| If you sign an Advance Directive (AD), you worry about being abandoned from treatment. |  | 2.60 | | 0.92 |
| Signing an Advance Directive (AD) is unnecessary, as you trust that your loved ones will make the right decisions on your behalf. |  | 2.71 | | 0.90 |
| You believe that even if you sign an Advance Directive (AD), you cannot be certain that the medical team will follow your wishes when needed. |  | 2.85 | | 0.84 |
| You are concerned that the signed Advance Directive (AD) may not cover all future medical decisions. |  | 2.87 | | 0.82 |
| Signing an Advance Directive (AD) makes you worry about reducing medical interventions by the healthcare team during your end-of-life period. |  | 2.66 | | 0.86 |
| You have already signed a Do Not Resuscitate (DNR) order or an Advance Medical Directive for Palliative and Life-Sustaining Treatment, so you do not need to sign an Advance Directive (AD) again. |  | 2.67 | | 0.74 |
| For you, signing a healthcare proxy is unnecessary as you don't want to burden your family. |  | 2.81 | | 0.89 |
| You don't believe that your current dialysis poses a threat to your life, so you have not considered topics related to death. |  | 2.72 | | 0.87 |
| You believe that for end-stage kidney (uremia) patients, prolonging life treatments are more important than opting for life-sustaining treatment. |  | 3.03 | | 0.95 |
| **PSDA (13-65)** | **75.77** | **45.46** | | **6.04** |
| The Patient Autonomy Act permits patients to exercise their right to refuse medical treatment, which pertains to natural death rather than euthanasia. |  | 3.34 | | 0.75 |
| Patients have the right to be informed about their medical condition, treatment options, potential outcomes, and risks, and have the right to choose and decide on the medical options provided by physicians. Others should not obstruct this process. |  | 3.50 | | 0.68 |
| During patient visits, the medical team should appropriately inform the patient about their medical condition, treatment strategies, interventions, medications, prognosis, and possible adverse reactions. Information may be shared with family members or relevant individuals only if the patient does not object. |  | 3.42 | | 0.76 |
| When engaging in Advance Care Planning (ACP), the patient, at least one first-degree relative, and the appointed healthcare proxy must all participate. |  | 3.54 | | 0.62 |
| Signing an Advance Directive (AD) requires prior engagement in Advance Care Planning (ACP) with the medical team, verification by the medical institution's seal, notarization by a notary public, or witnessing by two or more individuals. It also needs to be noted on the National Health Insurance (NHI) IC card to become effective. |  | 3.54 | | 0.62 |
| Even after signing an Advance Directive, you can still withdraw or modify it in writing at any time. |  | 3.63 | | 0.63 |
| If the patient's most recent medical decision is to receive life-sustaining treatment or artificial nutrition and hydration, the physician should immediately carry out the patient's wishes. |  | 3.55 | | 0.61 |
| If the patient's most recent medical decision is to decline life-sustaining treatment or artificial nutrition and hydration, the physician should still adhere to the original Advance Directive until the IC card notation change process is completed. |  | 3.41 | | 0.69 |
| The appointed healthcare proxy must be at least twenty years old, possess full legal capacity, and their appointment should be in writing. |  | 3.67 | | 0.60 |
| The healthcare proxy can represent the patient's medical wishes when the patient is unconscious or unable to communicate clearly. This includes receiving medical information from the medical team, signing consent forms, and expressing the patient's medical wishes based on the content of the patient's Advance Directive. |  | 3.61 | | 0.60 |
| If there are multiple healthcare proxies appointed, they can individually execute the patient's Advance Directive without the need for all of them to be present. |  | 3.43 | | 0.67 |
| If a patient meets clinical conditions such as being in an end-stage state, irreversible coma, permanent vegetative state, severe dementia, and has an Advance Directive, the medical team may terminate, withdraw, or not implement all or part of life-sustaining treatment or artificial nutrition and hydration based on the patient's Advance Directive. |  | 3.62 | | 0.63 |
| Based on their expertise or preference, the medical team reserves the right to not carry out the patient's Advance Directive. |  | 3.21 | | 0.80 |
| **Importance (10-50)** | **77.62** | **38.81** | | **6.83** |
| Enhance patients' and families' understanding of the disease, including prognosis, treatment choices (dialysis treatment, conservative treatment, and palliative care services), and potential outcomes of these treatment options. |  | 3.92 | | 0.69 |
| Guide patients in determining care goals, including scenarios involving loss of capacity and end-of-life situations. |  | 3.90 | | 0.70 |
| Collaborate with patients to develop care plans that align with their goals, incorporating their preferences for situations involving loss of capacity, specific clinical conditions, and end-of-life care. |  | 3.88 | | 0.72 |
| Ensure that healthcare professionals follow the patient's Advance Directive (AD) when making medical decisions. |  | 3.84 | | 0.73 |
| Help patients ensure the quality and dignity of their lives, maintaining emotional tranquility. |  | 3.91 | | 0.76 |
| Facilitate opportunities for patients and their loved ones to discuss the patient's future care preferences. |  | 3.92 | | 0.73 |
| Assist in alleviating the emotional burden that patients and their loved ones experience when making medical decisions. |  | 3.86 | | 0.73 |
| Support patients in designating healthcare proxies for future care decisions. |  | 3.83 | | 0.76 |
| Assist healthcare proxies in understanding their roles and responsibilities in the patient's future medical decisions. |  | 3.83 | | 0.75 |
| Promote mutual understanding of the patient's values and wishes among the patient, healthcare proxies, and healthcare professionals. |  | 3.90 | | 0.74 |
| **Willingness** | **Score** | | **Mean** | **SD** |
| Stated intention (7-28) | 69.52 | | 19.47 | 4.04 |
| Are you willing to sign an "Advance Directive" (AD)? |  | | 2.75 | 0.65 |
| Are you willing to sign an "Advance Directive" (AD)? |  | | 2.77 | 0.67 |
| Are you willing to encourage your family members to participate in Advance Care Planning (ACP) as well? |  | | 2.77 | 0.63 |
| Are you willing to encourage your family members to sign an "Advance Directive" (AD) as well? |  | | 2.74 | 0.61 |
| Are you willing to sign a "Do Not Resuscitate (DNR) Order or Advance Medical Directive for Palliative and Life-Sustaining Treatment"? |  | | 2.78 | 0.66 |
| Are you willing to sign an "Advance Healthcare Proxy Directive" to designate a healthcare proxy? |  | | 2.78 | 0.60 |
| Are you willing, under a physician's recommendation, to reduce or terminate dialysis during end-of-life and specific clinical conditions? |  | | 2.88 | 0.61 |
| Willingness for Life-Sustaining Treatment (5-20) | 54.05 | | 10.81 | 2.90 |
| If diagnosed with specific clinical conditions one day, would you be willing to undergo "nasogastric tube" feeding treatment? |  | | 2.02 | 0.77 |
| If diagnosed with specific clinical conditions one day, would you be willing to undergo "tracheal intubation" treatment? |  | | 1.88 | 0.70 |
| If diagnosed with specific clinical conditions one day, would you be willing to receive "Cardiopulmonary Resuscitation" (CPR) emergency measures? |  | | 2.09 | 0.76 |
| If diagnosed with specific clinical conditions one day, would you be willing to receive "emergency medication injections" emergency measures? |  | | 2.12 | 0.72 |
| If diagnosed with specific clinical conditions one day, would you consider stopping or reducing dialysis? |  | | 2.69 | 0.73 |
| **Other related questions** regarding ACP and PSDA | N | （%） | | |
| "Most" wanted to discuss your end-of-life wishes with whom? |  |  | | |
| None | 16 | 12.4 | | |
| Healthcare professionals | 22 | 17.1 | | |
| Family members | 88 | 68.2 | | |
| "Most" preferred timing for Advance Care Planning (ACP): |  |  | | |
| When healthy | 61 | 47.3 | | |
| When kidney function starts to decline | 19 | 14.7 | | |
| When kidney function deteriorates and dialysis is needed | 11 | 8.5 | | |
| When other severe complications arise | 24 | 18.6 | | |
| When in a life-threatening situation | 9 | 7.0 | | |
| "Most" appropriate initiator of Advance Care Planning (ACP): |  |  | | |
| The patient themselves | 50 | 38.8 | | |
| Nephrologists (kidney specialists) | 67 | 51.9 | | |
| Others | 8 | 6.2 | | |
| Whom you want to designate as your healthcare proxy: |  |  | | |
| Spouse | 72 | 55.8 | | |
| Children | 64 | 49.6 | | |
| Siblings | 17 | 13.2 | | |
| Friends | 2 | 1.6 | | |
| Others | 15 | 11.6 | | |
| How much are you willing to pay out-of-pocket for participating in Advance Care Planning (ACP)? (US dollars) |  |  | | |
| 33 | 93 | 72.1 | | |
| 66 | 9 | 7.0 | | |
| 99 | 13 | 10.1 | | |
| 165 | 2 | 1.6 | | |
| 197 | 2 | 1.6 | | |
| 263 | 1 | 0.8 | | |
| Believe that the "Advance Care Planning (ACP) consultation fees" should be paid by whom: |  |  | | |
| Pay in full by oneself | 11 | 8.5 | | |
| Fully covered by National Health Insurance | 59 | 45.7 | | |
| Fully subsidized by the government | 29 | 22.5 | | |
| Fully funded by a charitable foundation | 3 | 2.3 | | |
| Shared equally between oneself and relevant parties | 27 | 20.9 | | |

S Figure 1. Research Framework

**Knowledge** of ACP, AD and PSDA

**Attitude** of ACP, AD and PSDA

**Willingness** of Knowledge of ACP, AD and PSDA

**Baseline data**

- Demographic data
- Dialysis
- Other experience
